# Adapting A Project-Based Aging Laboratory During a Pandemic

**DOI:** 10.1093/geroni/igab046.408

**Published:** 2021-12-17

**Authors:** Troy Andersen, Linda Edelman

**Affiliations:** 1 University of Utah, University of Utah College of Social Work, Utah, United States; 2 University of Utah College of Nursing, Salt Lake City, Utah, United States

## Abstract

Aging Well in Utah is a competitive 2-semester project-based Honor’s College Praxis Laboratory for 9 students from different degree programs dedicated to deepening understanding of the aging process through a broad gerontological lens. This session will address how the course was adapted during the COVID-19 pandemic, including: 1) scheduling virtual class times with guest lecturers; 2) conducting older adult interviews via Zoom to provide students experience in communicating “what matters most”, one of the 4Ms of Age Friendly HealthCare; 3) adapting a student-designed medical narrative project highlighting stories of transition and healing through the dying process for previously unsheltered residents of a hospice program to focus on the lived experience of hospice patients during COVID-19; and 4) utilizing virtual technology to interview hospice patients, family members and hospice staff. In spite of challenges, student evaluations were above average and reported increased interest in incorporating age-friendly concepts into future careers.

